# Supplementary material for: Are carbon emissions trading and green financial instruments synergistic? -Comprehensive quantitative research based on content analysis
Source: PLoS One. 2024 Mar 7;19(3):e0298601. doi: 10.1371/journal.pone.0298601 (PMC10919601; doi:10.1371/journal.pone.0298601)
Supplement: S1 Data — (ZIP) [file pone.0298601.s001.zip › Data/地级市Z_F工作报告.zip!/╜¡╦╒/╬▐╬2╩╨/└┤╫╘╣1⁄2╓┌║┼í╛┬φ┐╦╩2╛▌═°í┐_2014.docx]

各位代表：

　　现在，我代表无锡市人民政府向大会报告工作，请予审议，并请市政协委员和其他列席人员提出意见。

　　2013年工作回顾

　　2013年是全面贯彻党的十八大精神的开局之年。一年来，面对错综复杂的经济环境，在中共无锡市委的正确领导下，全市上下认真贯彻落实中央和省委省政府决策部署，紧紧围绕科学发展主题和加快转变经济发展方式主线，积极应对各种困难挑战，统筹做好稳增长、促转型、惠民生各项工作，经济社会保持平稳发展的良好势头。

　　（一）经济运行稳中有进

　　坚持科学发展不动摇，认真按经济规律办事，充分发挥企业主体作用和政策引导作用，切实加大有效投入，挖掘消费潜力，开拓国际市场，全力推动经济稳定健康发展，努力使经济运行保持在合理区间。预计地区生产总值增长9.3%，公共财政预算收入增长8%，固定资产投资增长18.1%，社会消费品零售总额增长12.5%，进出口总额、出口总额与上年基本持平。加大对企业的帮扶和减负力度，强化要素协调保障，完善“稳心留根”办法，取消或免征涉企收费74项，降低收费标准26项，实现社会融资总量1921亿元，其中新增人民币贷款641.1亿元，新增境外上市公司2家，10家企业在区域产权交易所挂牌。坚持用市场手段妥善处理金融风险，经过大量艰苦细致的工作，尚德公司、申达集团成功实现破产重组，光伏、钢贸等领域不良贷款处置取得重大进展。

　　在全力稳增长的同时，切实把工作的着力点放到提高经济发展的质量和效益上来，出台一系列政策意见，强化集约节约发展导向，深入开展“项目建设提升年”活动，着力推进千企转型升级，实施“百企做强、千企技改”行动计划，开展“百企百行”金融挂钩活动，经济发展的质量进一步提高。预计全市规模以上工业企业利润增幅较上年提高10个百分点左右。14家企业入选中国企业500强，21家企业入选中国制造业企业500强。万达文化旅游城、天朗无锡运河古城、海力士增资等一批投资超百亿的重大项目成功签约。全市单位地区生产总值能耗下降4.1%，预计亿元地区生产总值占用建设用地量下降7.2%。无锡再次获评福布斯“中国大陆最佳商业城市”，被评为全国质量强市示范市、全国国土资源节约集约模范市、国家电子商务示范城市，兴澄特钢获“省长质量奖”。

　　（二）转型升级步伐加快

　　围绕苏南现代化示范区建设目标，着力打造“三地三中心”，构建现代产业体系。加大传统制造业改造升级力度，壮大新兴产业规模，推动制造业提质增效，预计全市高新技术产业产值占规模以上工业总产值比重较上年提高1.9个百分点，新兴产业产值增长14%左右。国家传感网创新示范区建设成效显著，物联网产业产值增长40%，新区跻身首批“智能传感系统创新型产业集群”。实施服务业发展超越工程，不断提升传统商贸优势，大力发展现代服务业，加快推进电子商务发展，预计服务业增加值占地区生产总值比重较上年提高0.8个百分点，恒隆广场、苏宁广场、百联奥特莱斯广场等一批城市商贸综合体开业，无锡（国家）数字电影产业园正式启用，预计全市接待境内外游客达7000万人次。加快建设国家现代农业示范区，设立现代农业发展基金，农业园区化面积比重较上年提高8个百分点，列入首批全国农业农村信息化示范基地，农业基本现代化水平居全省前列。

　　深入实施创新驱动和人才优先发展战略，全面推进国家创新型城市试点工作，加快智慧城市建设，积极集聚各类科技创新创业要素，全市新增“530”企业88家，新引进5名诺贝尔奖获得者和8名外国院士来锡建立工作站和研究院。全社会研发投入占地区生产总值比重达2.7%。万人有效发明专利拥有量达14.8件，比上年提高3.85件。江阴高新区成功创建国家级创新型特色园区。无锡蝉联中国智慧城市发展评估第一名，列入“国家下一代互联网示范城市”，入选国家知识产权示范城市，连续第十次被评为“全国科技进步先进市”。

　　（三）改革开放活力增强

　　围绕形成有利于科学发展的体制机制，实施重点领域和关键环节改革。狠抓简政放权工作，国家和省要求取消或下放的334项行政审批事项全部落实到位。推进行业组织与主管单位“三脱钩”，推动政府相关职能向行业组织转移，扩大政府购买公共服务范围。深化投融资体制改革，出台政府性项目投融资管理制度，健全投融资工作机制和政府负债预警机制，实现惠山古镇融资平台债务重组。加快国有资本布局调整，组建无锡文旅集团、江苏资产管理公司、无锡金融投资公司，推进东航江苏公司无锡分公司建设。鼓励和引导民间资本进入基础设施、社会事业等领域，累计19个项目成功与民企签约。深入推进农村产权制度改革，农村集体土地所有权确权登记发证工作全面完成并实现全覆盖，加快集体经济股份合作制改造，帮扶经济薄弱村发展，全市村均集体收入达660万元，继续保持全省领先。积极开展省“两型社会”综合配套改革试点，完善环境资源区域补偿机制，无锡列入全国绿色低碳交通区域性试点城市、全国节水型社会建设示范区和国家城镇低效用地再开发试点城市。

　　围绕构建开放型经济体系，进一步扩大对外开放。全面对接上海自贸区建设，积极申报和探索建设空港型自由贸易园区。苏南硕放国际机场全面对外开放取得突破，成功开通至新加坡国际航线，境外航线增加至9条，国内航线增加至34条，机场旅客吞吐量达359万人次，同比增长10.9%。推动开发区转型升级，宜兴经济开发区升级为国家级开发区。利用外资在困难环境中继续前进，实际到账外资33.4亿美元。“走出去”步伐继续加快，全市新批境外投资规模居全省第二。加强国际友好交流，国际友城增加到46个，无锡和美国友城圣安东尼奥市同获首届中美友好城市“最佳创新奖”。成功举办首届全球锡商大会。

　　（四）城乡建设协调发展

　　坚持统筹城乡发展，构建优美宜居、产城融合的现代化城乡新格局。制定实施“一城一岛一带”规划建设三年行动计划，太湖新城、马山国际旅游岛、古运河风光带建设步伐加快。江阴临港新城、宜兴东氿新城、锡东新城、惠山新城等重点区域开发成效显著。启动10个城乡一体化先导示范镇建设，进一步开展“三集中两置换一转化”工作，全市农业向适度规模经营集中比例达85%，乡镇工业向园区集中比例达91%，农村居住集中比例达45%，城乡一体化发展取得新的进展。无锡获评中国内地宜居城市竞争力第一名。

　　加强城市基础和配套设施建设。地铁1号线即将进入试运行，2号线全线“轨通”，第二轮轨道交通建设规划获批。宁杭高铁无锡段建成通车，苏南硕放国际机场二期改扩建工程基本建成。刘闾路、杨胡路等市区道路建成通车，市区新辟、调整公交线路39条，新购公交车273辆。国内首条安全供水高速通道贯通投用。改造完成自来水老旧管网84.1公里，新增天然气总用户6万户。建成4G基站1406个、免费无线接入点6775个。

加强城乡环境综合整治。完成中心城区城中村改造32万平方米，旧住宅区整治200多万平方米，危旧房改造20万平方米，建成一批城中村环境整治改造样板点。严格实行依法征收，完成房屋征收483万平方米。实施新型城镇化和村庄环境整治行动计划，全市9079个自然村环境整治全部完成，规划保留村庄全部达到二星级以上康居乡村标准。美丽乡村建设取得明显成效，江阴华西村、宜兴张阳村等5个村庄入围“全国美丽乡村”试点，阳山镇桃源村被评为“中国最有魅力休闲乡村”，周铁镇荣获中国人居环境范例奖。

（五）生态文明不断进步

　　加强环境保护和污染治理，突出抓好太湖治理、大气防治、污染减排、植树增绿、环境执法等重点工作。强化太湖长效治理和应急防控，推进重点治太工程建设，太湖无锡水域未发生大面积湖泛，确保了饮用水安全，连续六年实现太湖安全度夏。顺利通过水利部全国水生态系统保护与修复试点验收，成为全国首批“水生态文明城市建设试点市”。实施新一轮“蓝天工程”，推进工业废气治理，开展机动车尾气污染防治和建设工地扬尘专项整治，落实总量减排措施，划定高污染燃料禁燃区，无锡西区燃气热电项目启动建设。启动147个日常生活垃圾分类收集试点。严厉打击各类环境违法行为，取缔关闭企业92家，停产整顿136家。化学需氧量、氨氮、二氧化硫、氮氧化物等四项主要污染物完成年度减排任务。着力优化生态空间，全市28.7%的土地划定为生态红线保护区域，严格落实耕地保护制度，全市基本农田稳定保持在165万亩。林木覆盖率和建成区绿化覆盖率分别达25.6%和42.7%，市区人均公园绿地面积14.6平方米。蠡湖湿地公园成为华东地区面积最大的开放式国家湿地公园。无锡荣获首批“国家生态市”称号，建成全国首个生态城市群。

　　（六）民生工作扎实推进

　　始终坚持民生优先，构筑民生“六大体系”，将本级财政支出的70%以上用于保障和改善民生，全面完成12件为民办实事项目。做好农村转移劳动力、高校毕业生等重点群体就业工作，健全城乡统一的就业创业服务体系，全市城镇新增就业14.8万人，城镇登记失业率2.12%，无锡被评为全国创业先进城市。积极拓宽城乡居民增收渠道，预计全市城镇居民人均可支配收入、农民人均纯收入分别增长9%和10.5%左右。稳步推进社会保障扩面提标，城乡基本养老、医疗、失业保险覆盖率均达98%以上，市区居民基础养老金每月提高到310元，企业退休人员养老金每月提高到2032元，市区城乡低保标准每月提高到600元，全市月最低工资标准提高到1480元。全面实施城乡居民大病保险制度，职工医保、居民医保政策范围内住院费用补偿率分别提高到80%和70%以上。新型农村合作医疗平均筹资达533.9元，江阴率先在国内建成基本医保、大病救助、补充保险“三位一体”的新农合保障体系。拓展“慈福民生系列”保险领域。提高社会办养老机构补助标准，失能老人托养中心、滨湖区社会福利院等建成投用，每千名老人拥有机构养老床位32张。无锡列入国家残疾预防综合体系试点城市，在全国率先建立综合性残疾人数字图书馆，崇安区残疾人综合服务中心启用。完善住房保障制度，全市新开工各类保障性住房11992套，市区廉租房在保家庭4433户。全力保障市场供应，加强蔬菜基地建设，推进平价商店建设和放心粮油连锁经营，居民消费价格上涨2.1%，物价水平保持稳定。

　　（七）社会事业成效显著

　　努力办好社会事业，提高公共服务能力。完善现代化教育体系，新建、改扩建幼儿园47所，率先全面建成“江苏省学前教育改革发展示范区”，80%的义务教育学校达到省现代化办学标准，各市（县）区全部首批通过“义务教育发展基本均衡县（市、区）”国家认定。深化职业教育综合改革，组织实施28个省现代职教体系建设试点项目。启动与江南大学新一轮合作共建，支持无锡太湖学院等民办高等院校创新发展。深入开展健康城市建设，稳步推进公立医院综合改革试点，深化公立医院与社区卫生服务机构协作，基层医疗卫生服务能力逐步提高，新增10个国家、省级示范社区卫生服务中心，居民在基层就诊比例提升至50%以上。率先建成省级慢性非传染性疾病综合防控示范区，有力防控人感染H7N9禽流感突发疫情。新中医院建成启用，江阴、锡山新医疗中心等启动建设，无锡市儿童医院通过国家三级医院定级验收。免费孕前优生健康检查和独生子女父母一次性奖励政策实现全覆盖。积极参与大运河申报世界文化遗产，加大力度推进惠山古镇祠堂群申遗，新增全国重点文保单位16处，荣巷、荡口历史文化街区开街，公共文化设施实行全年无休免费开放。吴都阖闾城遗址入选第二批国家考古遗址公园。成功举办世界青年射箭锦标赛、环太湖国际公路自行车赛及第十一届市运会等赛事，无锡成为全国首批、省内唯一全国全民健身示范城市。

　　（八）社会管理持续加强

　　创新社会管理理念、体制、机制，完善多方参与的社会管理体系，和谐社会建设取得新成效。提升基层管理能力，全市村、居委圆满完成新一届换届选举工作，按照“一委一居一站一办”服务管理架构运行。推进平安无锡建设，全市80个镇、街道均成立政法综治工作中心。深化国家级流动人口社会融合试点，无锡连续荣获“全国社会管理综合治理优秀市”。全面开展安全生产大检查，加强事故隐患排查治理体系和企业安全生产标准化建设，全市安全生产事故死亡人数同比下降10.3%。加强食品、药品安全监管，食品安全和药品检测合格率分别达到97.4%、98.6%，肉菜追溯体系覆盖率达80%。健全突发公共事件工作网络，完善应急处置总体预案和专项预案，应急处置能力有效提升。完善大调解机制，依法依规处理信访突出问题，一批征地拆迁、劳资纠纷、环境保护等信访积案得到有效化解。全面开展第三次全国经济普查工作。史志、档案、侨务、台港澳、外事、民族宗教、人武、人防、双拥、气象、防汛防台、防震减灾等工作取得新成绩，妇女、儿童、红十字、老龄、残疾人等事业有了新发展。

　　去年以来，市政府切实加强自身建设，确保各级行政管理机关依照法定权限和程序行使职权、履行职责。认真贯彻市人民代表大会及其常委会的各项决议，扎实办理人大代表的建议、批评、意见和政协委员的提案、建议案。加强市“12345”政府公共服务平台规范化建设，推进行政权力网上公开运行，实施“六位一体”评议政风行风创新行动，创立全媒体问政栏目“政风面对面”。认真执行中央八项规定、省和市委十项规定要求，落实党风廉政建设责任制。充分发挥审计监督作用，严格预算管理，严肃财经纪律，进一步控制和压缩财政性一般经费支出，全市“三公”经费财政拨款预计执行数较上年决算数同比下降30%。深入开展“作风建设提升年”活动，切实改进机关作风，提高行政效能，服务发展水平有了新提升。

　　各位代表，过去一年，无锡经济社会发展取得的成绩来之不易，凝聚着全市人民的智慧和汗水。在此，我代表无锡市人民政府，向在各个领域和不同岗位上辛勤劳动、无私奉献的全体市民，向辛勤工作在村组、社区、企业等基层组织和单位的广大干部员工，向给予政府工作积极支持的人大代表和政协委员，向离退休老同志，向各民主党派、工商联、各人民团体和各界人士，向中央及省在锡单位，向驻锡人民解放军、武警官兵和公安干警，向所有参与、支持和关心无锡建设发展的海内外朋友，表示崇高的敬意和衷心的感谢！

　　各位代表，我们也清醒地看到，我市经济社会发展中还存在一些问题和矛盾，政府的工作也存在一些不足之处。经济增长的基础还不够牢固，一些中小企业生产经营困难，少数经济指标虽经努力仍未达到预期目标；新引进重大产业项目还不多，新兴产业对经济增长的支撑力不强；光伏、钢贸等行业金融风险尚未彻底消除，政府债务风险不容忽视；环境保护工作任务艰巨，太湖水和空气质量有待进一步改善；有些政府部门和工作人员主动服务和积极作为意识不强，履职效率和服务质量有待提升，反腐倡廉建设力度需要进一步加大。我们将对此高度重视，在今后的工作中努力加以解决。

　　2014年工作任务

　　今年是深入贯彻落实党的十八届三中全会精神、全面深化改革的第一年，也是深入推进苏南现代化示范区建设、完成“十二五”规划目标任务的关键一年。做好今年政府工作的总体要求是：全面贯彻落实党的十八大和十八届二中、三中全会精神，按照省委、省政府和市委的决策部署，紧紧围绕率先基本实现现代化总体目标，坚持稳中求进工作总基调，把改革创新贯穿于经济社会发展各个领域各个环节，着力深化全面改革开放，着力推动经济转型升级，着力加强生态文明建设，着力发展民生社会事业，着力解决好经济社会发展中存在的矛盾和问题，进一步激发市场活力、筑牢产业优势、优化宜居环境、增进民生福祉，推动“四个无锡”建设迈上新台阶。

　　今年经济社会发展主要预期目标是：地区生产总值增长9%左右；公共财政预算收入增长8.5%左右；城乡居民收入增长与经济增长同步；城镇登记失业率控制在3.5%以内；居民消费价格指数控制在省定范围内；万元地区生产总值能耗和主要污染物排放削减量完成省下达目标。这些指标的安排，适应了国内外形势的新变化，结合了无锡发展实际情况，既考虑了稳增长的要求，也为深化改革、转型升级和改善民生留出了空间，有利于引导各方把经济工作重点放到稳中求进、改革创新的核心要求上来。

做好今年工作，必须坚持稳中求进的工作总基调。在当前经济下行压力较大的形势下，坚持科学发展仍然是解决所有问题的关键这一重大战略判断，千方百计保持经济稳定增长，努力实现经济发展质量和效益得到提高又不会带来后遗症的速度。认真贯彻落实积极的财政政策和稳健的货币政策，推动各项结构性减税政策落实到位，积极争取上级政策性引导资金支持，更好地发挥地方财政引导资金的杠杆效应。加大金融服务实体经济力度，继续着力化解和压缩不良贷款，积极破解企业资金链风险难题，深入开展“百企百行”金融挂钩活动，努力实现全社会融资总量超2000亿元，其中新增本外币贷款超800亿元。大力推动企业公开上市和挂牌交易，力争新增公开上市和挂牌交易企业30家。不断完善“稳心留根”办法，进一步强化要素保障，支持企业稳定发展。充分发挥消费的基础作用，积极培育和拉动信息、文化、休闲、养老、健康和家居服务等消费热点，努力实现社会消费品零售总额增长13%左右。充分发挥投资的关键作用，全力以赴争取和引进重大项目，全年安排市级重点项目180项，总投资4610亿元，年内计划投资1000亿元，全社会固定资产投资增长16%，争取实现到位注册外资38亿美元。充分发挥出口的支撑作用，奋力开拓新兴国际市场，巩固传统优势市场，努力实现进出口总额、出口总额适度增长。

　　在稳增长的基础上，今年重点做好以下五个方面工作：

　　（一）全面深化改革开放激发创业无锡活力

　　紧紧抓住全面深化改革带来的重大机遇，大力推进改革创新，尊重基层首创精神，以更大决心冲破思想观念的障碍、突破利益固化的藩篱，为各类市场主体营造宽松有序发展环境，让一切劳动、知识、技术、管理、资本的活力竞相迸发，让一切创造社会财富的源泉充分涌流，让一切创业主体的积极性空前高涨，为新形势下的无锡发展打开新空间。

　　深化行政审批制度改革。进一步简政放权，落实好国家和省取消和下放的审批事项，继续取消和下放一批市级行政审批事项。建立各部门行政审批事项目录清单制度，并向社会公开。大力推进工商注册制度便利化，变注册资本实缴登记制为认缴登记制，放宽企业住所或经营场所的限制。加快建立行政审批、为民服务、公共资源交易一体化的综合政务服务体系。理顺责权关系，建立事权和支出责任相适应的财税制度，研究更加合理的市、区财税体制，完善乡镇、街道财力保障机制，增强基层政府公共服务能力。

　　深化经济体制改革。把经济体制改革作为全面深化改革的重点，使市场在资源配置中起决定性作用和更好发挥政府作用。放宽民间资本市场准入，消除各种隐性壁垒，支持和鼓励民间资本参与基础类、公益类项目建设和国有企业改革，支持和鼓励设立民营银行和其他民营金融机构。完善国有资产管理体制，改革国有资本经营体制，健全考核激励机制，增强国有企业市场竞争力。支持和鼓励国有资本投资项目吸收非国有资本参股，发展混合所有制经济。大力推动金融业改革创新，做大做强做优银行、证券、信托、财务公司、资产管理等地方法人金融机构，加快组建国联寿险公司，争取组建金融租赁公司、消费金融公司，积极发展各类股权投资基金，探索发展网络金融平台，深化农村金融改革，积极发展村镇银行、小额贷款公司。扎实推进滨湖区省级服务业综合改革试点。积极发展技术市场，健全技术转移机制，完善风险投资机制，促进科技成果资本化、产业化。健全城乡发展一体化体制机制，继续推进村（社区）经济股份合作制改革，深化农村土地征收制度改革，完善农村土地承包经营权流转制度，引导农村住宅和承包土地依法自愿有偿有序流转、转让，试点开展农村产权抵押、担保和交易，赋予农民更多财产权利。创新构建新型农业经营体系，提升新型农村合作经济组织发展水平。

　　深化社会领域改革。研究符合城市规模和承载力的人口政策，促进产业、城镇的合理布局和有效集聚。启动实施一方是独生子女的夫妇可生育两个孩子的政策。全面实施县级公立医院改革，探索市级公立医院改革新模式，完善分级诊疗体系，优化社区家庭医生与居民契约服务机制。健全义务教育均衡发展长效机制，完善中小学教育质量科学评价体系，按照国家和省统一部署做好招生考试制度改革工作，充分发挥教育督导作用。加快建立社会化的早教和养老服务体系，鼓励民间资本投资建设高水平的学校、医院和养老机构。进一步推进社会事业“管办分离”，理顺职能分工，做到“管”得更科学公正，“办”得更规范高效。加快推进事业单位分类改革。创新社会治理体制，完善社会组织管理制度，降低社会组织准入门槛，加大政府职能向社会组织让渡力度，探索在城市管理、环境保护、社会管理等领域充分发挥社会组织作用。

　　进一步扩大对外开放。抢抓上海自贸区发展机遇，做好无锡高新区综保区、空港产业园区、江阴港区等与上海自贸区的政策接轨，提升服务功能。创新开发区管理体制机制，力争在口岸监管、贸易开放、人才引进、利用外资等方面取得突破。积极推进惠山经济开发区、江阴临港经济开发区申报国家级开发区，争取江阴保税物流中心升级为综合保税区。积极引进外资企业总部和功能性机构，引导外资更多投向高新技术产业、战略性新兴产业和现代服务业。加大国际市场开拓力度，优化外贸出口结构，落实信用保险等政策，提升重点出口基地发展水平，扩大自主品牌产品和服务贸易出口比例。鼓励企业“走出去”投资设厂、建立分销网络，有效规避贸易壁垒，扩大国际市场占有率，支持柬埔寨西港特区加快发展。努力提高城市国际化水平，建设一批国际化的教育、医疗、体育和文化设施，建立与国际人才流动相适应的社会保障制度，完善城市多语种服务体系，积极构建国际化的人居环境、生态环境和服务环境。加强国际经贸文化交流，借助国际友城等资源，拓展国际交往渠道，扩大城市推介和形象宣传，进一步提升无锡城市的国际影响力。

　　（二）全力推进经济转型升级增添创新无锡动力

　　坚持把转方式、调结构、抓创新作为克服经济困难和矛盾、促进经济持续健康发展的治本之策。无锡之所以能够赢得民族工商业发祥地、乡镇企业摇篮和百年工商城等盛誉，靠的是深厚吴文化孕育的实力企业、驰名产品和先进技术。面对经济下行压力和产能过剩矛盾，必须充分发挥自身优势，以更大决心冲破各种困难险阻、推动经济转型升级，实现凤凰涅槃、浴火重生，让深厚的吴文化底蕴成为培育自主创新的肥沃土壤，让优美的宜居环境成为做大做强企业的丰富资源，让百年工商城的盛誉成为打造驰名品牌的雄厚基础，为新形势下的无锡发展增添新动力。

　　大力发展先进制造业。实施领军企业家塑造计划，着力发掘和培育一批秉承“锡商”优良传统、具有国际眼光和现代企业管理水平的优秀企业家，使之成为引领企业做强做大、推进经济转型升级的领头雁。实施品牌发展战略，着力扶持和培育一批具有良好市场基础、先进技术和丰富文化内涵的自主品牌，使之成为提升无锡产品市场竞争能力、提高经济质量和效益的突击队。鼓励本土企业外引内联，主动寻求与国有大企业开展资本合作、与国外先进企业开展技术合作，加强与国内产业链企业的兼并重组，通过资源整合与技术嫁接，实现企业裂变、跨越发展，争当国际国内行业排头兵。深入开展“项目建设深化年”活动，积极推进企业技术改造，全年开工和竣工1000万元以上工业投资项目1000个，其中亿元以上300个，紧盯先进制造业项目加强招商引资，实现工业投入增长15%，确保投资超10亿元重大项目有较大增长。大力推动物联网、微电子、新材料、节能环保、生物医药、石墨烯等新兴产业加速发展，实现新兴产业产值增长15%以上。争创中国软件名城。推动光伏产业适应性调整，实施分布式光伏发电计划，扩大光伏发电应用规模，重塑无锡光伏产业发展优势。加快建设宜兴、惠山经济转型集聚区，推动无锡经济开发区提档升级，提高各级各类经济开发区和工业园区产业承载能力。坚决化解过剩产能，加快淘汰落后产能，积极发展清洁生产、循环经济。

　　加快发展现代服务业。充分发挥无锡资源优势，完善激励政策，大力推动金融、会展、现代物流、大宗商品交易、旅游休闲、健康养老、商务中介、服务外包、文化创意、信息和大数据等现代服务业加快发展，争取服务业增加值占地区生产总值比重提高1个百分点。聚焦服务业重点平台和载体建设，增强其辐射带动效应。积极争创海峡两岸金融与科技合作试验区，大力吸引中外资各类金融机构入驻。加快建设万达文化旅游城、天朗无锡运河古城等高端旅游文化综合体，加快推进徐霞客旅游度假区、阳羡湖旅游小镇、阳山东方田园等乡村游资源开发。推动无锡（国家）数字电影产业园、新区创意产业园、无锡国家动画产业基地等文化产业集聚区加速发展，创建国家对外文化贸易基地，争取设立文化产业保税区。加快建设“锡货网上行”、众瀛联合数据等网络平台，充分利用全国知名电子商务平台，争取开展跨境贸易电子商务服务试点。进一步推动大中型企业实施“主辅分离”，将研发、销售、物流等环节从生产链中分离出来，发挥资源整合效应，推动总部经济创新发展。

　　着力提高农业现代化水平。提升国家现代农业示范区建设水平，加快推进无锡现代农业博览园等农业园区发展，全市高标准农田面积比重提高到63%，新建千亩以上市级现代农业园区10个，农业园区化比重达40%。大力发展优质、高效、生态农业，重点培育发展生物育种产业，提升旅游休闲农业发展水平。加强耕地和基本农田保护，健全农田水利保障体系。强化粮食安全和应急体系建设，抓好“菜篮子”工程，加强蔬菜基地建设与管理，提高市场保供能力。

　　深入实施创新驱动战略。把人才引领作为创新驱动的核心，实施人才强企工程，全面推进“东方硅谷”建设。采取有效政策吸引和集聚科技创新创业领军人才和团队，柔性引进外国专家、海外智力为企业服务，加快培养中高级应用型技工人才，更好地满足企业各类人才需求。积极开展无锡国家创新型城市试点，健全产学研协同创新机制，优化各类科技创新载体、孵化器功能，突出企业创新主体地位，实施大中型工业企业研发机构全覆盖计划和专利申请全覆盖计划，多渠道增加科技投入，全社会研发投入占地区生产总值比重达到2.75%。倾力打造升级版“530”，深化与本土企业、金融资本的有效对接和战略合作，促进百家“530”企业做大做强，尽快形成规模效应。加快打造“智慧无锡”，高标准建设国家传感网创新示范区，强化顶层设计和资源共享，推广各类综合应用终端服务，实施一批应用效果突出、产业带动性强、技术关联度高的典型应用工程，抓好一批技术研发和产业化项目，提升城市智能化和物联网技术应用水平，形成无锡创新驱动的特色标志。新增免费无线接入点2.4万个，实现重要公共场所全覆盖。

（三）着力加强生态文明和城乡建设彰显魅力无锡特色

　　高度重视人民群众普遍关心的环境质量改善、城乡面貌优化问题，善于运用倒逼机制，拿出实招，力见实效，以更大决心推进铁腕治污和环境综合整治，切实将生态文明和文化内涵融入城乡建设全过程，努力打造传承历史文化、保留城市记忆、彰显生态之美的现代化滨水花园城市，增强无锡作为最具竞争力宜居城市的发展新优势。

　　加强生态文明建设。深入实施“两型社会”配套改革，加快形成节约资源和保护环境的产业结构和生产生活方式，创建国家生态文明建设示范市和水生态文明建设示范市。开展新一轮太湖治理，加快推进太湖新城、望虞河西岸、直湖港、宜兴太湖西岸四大片区环境综合整治和中心城区控源截污工程，实施竺山湖生态清淤、新沟河拓浚等工程，深入推行入湖河流“断面长制”管理，加强水质监测监控和饮用水源地保护，确保饮用水安全、确保不发生大面积湖泛。继续实施新一轮“蓝天工程”三年行动计划，完善监测预警体系，推进钢铁、水泥、热电、化工等行业专项整治，加强建设工地扬尘、机动车尾气等污染防治，推广秸秆全面综合利用，努力改善空气质量。加强生活环境整治，扩大生活垃圾分类收集试点，巩固提升农村环境连片整治成果。规划建设静脉产业园，完善城市生活垃圾无害化处理体系。深入开展重金属污染专项整治，推进土壤修复工程，加强固体废物规范化管理。实行最严格的环境保护制度，严肃查处、严厉打击偷排偷放等环境违法行为。优化能源消费结构，完善能源消耗强度与能源消费总量“双控”制度。完成造林绿化面积2万亩，林木覆盖率达到26%以上。坚持节约用地和节约用水，推进水价改革，建立对重点生态功能区的生态补偿制度，引导市民形成绿色生活和消费方式，完善覆盖全社会的环保宣传教育体系。

　　提高城乡建设水平。适应形势新发展新变化，修改完善城乡总体规划、控制性详规、城市主体功能区规划和新农村建设规划。积极推动锡澄宜“一体两翼”协调发展，加强锡澄宜一体化发展重大问题研究，搞好重大基础设施的布局规划，争取启动苏锡常南部高速无锡段建设，完善锡澄南北通道建设，开展一批连接周边市（县）及重点乡镇快速道路的前期工作。加强城市重大基础和公共设施建设，保质保量推进地铁工程建设，确保1、2号线如期安全投运，安排好与常规公交和非机动车有序换乘相关设施建设，做好3、4号线规划设计和前期准备工作；建成启用苏南硕放国际机场新航站楼，开通更多国际国内航线；优化城市路网，市区建成北中路互通、广石路、东安路和西环线等道路设施；扩大公交线路覆盖面，市区新辟、优化公交线路15条，新增和更新低碳环保公交车200辆，4G网络公交车覆盖率50%以上；完成老旧自来水管网改造60公里，实施解放环路市政公用管线改造；启动建设气象灾害预警中心。坚持老城改造与新城建设并举，推动老城区有机更新，实施背街小巷治理，开展社区环境连片整治，搞好中山路、人民路等重要道路的环境提升。坚持村镇建设与城区建设并行，全面推进以人为核心的新型城镇化，优化重点乡镇布局，推动功能设施完善和产业集聚，促进公共服务向村镇延伸，加快产城融合发展。做好规划保留村庄和民俗建筑的保护，建设具有江南风貌与地域特色的美丽乡村。完善城市管理行政执法体制机制，强化城市环境综合整治和精细化管理，提升执法和服务水平。高度重视征地搬迁工作，推进依法征收，为城乡建设和民生改善提供保障。

　　提升城市文化品质。坚持城市建设与历史文化融合发展，弘扬和彰显吴文化、山水文化和工商文化特色。突出“一城一岛一带”建设的文化塑造，使之成为城市新名片。精心打造太湖新城，着力完善文化、休闲、商业、学校、医院等配套服务，尽快实现金融商务街区、会展片区和沿湖滨水片区建设的重点突破，加速形成产业和人气集聚效应，加强蠡湖湾规划设计和开发工作，集中展示生态宜居和山水文化形象。精心打造马山国际旅游岛，充分利用吴文化品牌优势和独特的山水人文资源，积极建设国际化、生态化、特色化的休闲旅游度假胜地。精心打造古运河风光带，重点推进惠山古镇、清名桥特色夜经济区、沿河绿地景观工程建设和沿岸旧住宅外立面整治，加快配套功能开发，建设民俗特征鲜明、城市记忆深刻、工商文化浓厚的历史文化长廊。积极传播紫砂文化，建设海峡两岸紫砂文化交流基地。大力弘扬城市文化精神，以文化建设促进市民素质提升，创建全国文明城市。

　　（四）加快发展民生和社会事业擦亮幸福无锡品牌

　　牢固树立以人为本、民生优先理念，坚持把保障和改善民生作为改革发展的出发点和落脚点，坚持把加快发展社会事业作为保障和改善民生的重要抓手，以更大决心解决好人民群众最关心最直接最现实的利益问题，实现发展成果更多更公平地惠及全体市民，让广大群众真切感受到生活水平不断提高、社会不断进步，增强对“幸福无锡”的认同感、自豪感。

　　提高城乡居民生活水平。努力提高城乡居民收入，更多关心中低收入者和困难群体，拓宽就业、创业、投资、社保及帮扶等增收渠道。健全就业服务机制，办好大学生创业园，增强对优秀大学生的吸引力，实现3万名大学生在锡就业，新增城镇就业10万人。提高城乡居民和企业退休人员基本养老金标准，基本养老、医疗保险分别扩面6万人，失业、工伤和生育保险分别扩面4万人。完善社会养老服务体系，推进市福利中心二期等养老机构建设，新增机构养老床位3000张以上，为2万户以上社区老年人家庭提供居家养老援助服务。开展医疗保险定点护理院试点，推进医疗机构与养老机构合作。完善社会救助体系，实施无业三、四级精神（智力）残疾人生活救助，建成特需儿童早期干预中心。建立市区户籍居民住房财产保险制度。改善居民居住条件，市区新建、筹集保障性住房3000套以上、竣工2000套以上，发放廉租房租赁补贴1500户以上。完成旧住宅区综合整治100万平方米和中心城区危旧房改造10万平方米。加强安置房、经适房等社区的物业管理，逐步推进老小区电梯改造，提升物业管理水平。

　　繁荣发展社会事业。深入推进教育现代化，探索建立现代学校制度，加强师资队伍建设，强化素质教育，促进学生全面发展。优化发展学前教育，新建、改扩建优质幼儿园30所以上，创建市学前教育现代化镇（街道）15个。全面实施基础教育质量提升工程，90%的义务教育学校达到省现代化办学标准。推进普通高中优质特色发展，提高职业教育办学水平。大力发展高等教育，深化与江南大学合作共建，加强无锡太湖学院省市共建。积极支持各级各类民办学校创新发展，支持和鼓励中外合作办学。进一步提升公共卫生服务均等化水平，健全重大疾病防控和应急救治体系。着力提升社区医疗卫生机构服务水平，完善与二级以上公立医院协作机制。加快江南大学无锡医学院及其附属医院、新市五院（传染病医院）建设。推进卫生信息化平台整合和建设，实现健康档案、电子病历等卫生信息互联共享。扎实推进国家公共文化服务体系示范区创建。完成大运河无锡段申遗验收，加快推进惠山古镇祠堂群申遗工作。整合吴文化品牌资源，提升鸿山、阖闾城国家考古遗址公园影响力。实施全民健身工程，实现社区（村）“10分钟体育健身圈”全覆盖，建成西蠡湖慢游系统全民健身带，办好世界斯诺克无锡精英赛、环太湖国际公路自行车赛、国际马拉松赛等重大体育赛事，全力备战第18届省运会。进一步做好档案、史志等工作，促进社会事业全面发展。

　　创新和加强社会治理。强化全民法制教育，增强全民法治意识。发挥政府主导作用，鼓励和支持社会各方参与，实现政府治理、社会自我调节和居民自治良性互动。完善社区管理体制和服务网络，加强信息化社区建设，减少对基层评比达标考核，压缩台账范围，增强基层组织为民服务能力。创新流动人口和特殊人群管理服务机制，做好国家流动人口卫生和计划生育基本公共服务均等化试点工作。积极稳妥推进食品药品安全监管体制改革，进一步提高监管效能和应急处置能力。切实加强安全生产工作，强化企业主体责任、部门监管责任和属地管理责任，建立健全隐患排查治理体系和安全预防控制体系，严格安全生产考核与责任追究，坚决遏制重特大事故发生。加大信访问题排查和社会矛盾化解力度，健全及时就地解决群众合理诉求机制，深入开展领导干部接访下访活动，着力化解信访积案。创新社会治理方式，发挥行业协会、商会、慈善组织、义工志愿者组织等各类社会组织的桥梁纽带作用，拓宽社情民意表达渠道，加强网络等舆论引导和规范。认真做好台港澳、侨务、民族宗教等工作，发挥好台湾同胞、港澳同胞、海外侨胞、宗教团体等积极作用。加强双拥共建工作，深入开展国防教育，提高军转安置和优抚工作水平。强化社会治安综合治理，完善防控体系建设，严密防范、依法打击各类违法犯罪活动，建设法治中国先导区和平安中国示范区。

　　（五）切实回应社会关切建设人民满意政府

　　牢固树立执政为民理念，坚持把人民群众赞成不赞成、满意不满意作为衡量和检验政府工作的最终标准，以开展党的群众路线教育实践活动为强大动力，以更大决心推动政府职能加快转变，深入开展“作风建设深化年”活动，不断提高行政服务效能，努力建设为民、高效、法治、廉洁的人民满意政府。

　　切实转变作风。大兴密切联系群众之风，大兴求真务实之风，深入基层调查研究，掌握第一手情况和资料，坚持问题导向，提高工作的针对性和有效性。切实回应百姓关切，全力解决群众反映强烈的突出问题，着力办好12件为民办实事项目；切实回应企业期盼，尽力为企业减负松绑、排忧解难；切实回应基层干部呼声，努力为基层组织的工作营造良好环境，提供有力支持。严肃查处对群众、企业和基层组织的正当诉求不响应、不作为、久拖不决的行为。

　　提高服务效能。大力弘扬“四千四万”的苏南精神和“三创三先”的新时期江苏精神，切实提高行政服务的质量和效率，努力建设服务型政府。满腔热忱地为群众、企业、基层办事，做到能快尽快、能早尽早。加强向上级机关的汇报沟通，积极争取工作指导和政策支持。健全重大项目服务机制，对重大项目狠抓落实，一抓到底。积极推广电子政务，扩大网上办事范围，创新行政管理方式和手段。切实加强地方政府性债务管理，严格举债程序、控制总量增长、优化负债结构，坚决防范和化解地方政府性债务风险。

　　严格依法行政。强化法治观念，自觉接受人民代表大会及其常委会的法律监督、工作监督，自觉接受人民政协的民主监督，坚持重大事项尤其是重大决策出台前向人大报告和向政协通报制度。落实行政执法责任制，确保各级行政管理机关依法履行职责。切实维护司法公正，做到公正执法、有法必依、违法必究。落实政府工作科学民主决策机制，完善集体研究以及公示、听证和专家咨询论证等制度，建立决策后评估和纠错机制。加强政务信息公开，推进财政预算决算和“三公”经费向社会公开，接受社会监督和舆论监督。加强行政监督和审计监督，加大对审计发现问题的督促整改力度，建立健全责任追究制度。

　　坚持廉洁从政。深入开展党的群众路线教育实践活动，坚决反对形式主义、官僚主义、享乐主义和奢靡之风，加强反腐倡廉教育，在全社会形成反腐倡廉的浓厚氛围。深入贯彻落实党风廉政建设责任制，注重从源头上预防和治理腐败，严肃查处重点领域和关键环节的腐败案件，切实解决发生在群众身边的不正之风和腐败问题。深入贯彻落实中央、省关于厉行节约、反对浪费有关规定，强化预算管理，严格禁止新建楼堂馆所，严格控制机构编制，严格压缩“三公”经费支出，积极推进公共机构节能和节约型机关建设，把更多的物力、财力用在群众最迫切需要解决的问题上，以廉洁高效的形象取信于民。

　　各位代表，做好今年工作，任务艰巨、责任重大、使命光荣。让我们紧密团结在以习近平同志为总书记的党中央周围，在中共江苏省委、省人民政府和中共无锡市委的坚强领导下，开拓创新，锐意进取，求真务实，团结拼搏，为把无锡的明天建设得更加美好而努力奋斗！
